# Medical Fees and Under Bidding

**Published:** 1880-09

**Authors:** 


Medical Fees and Under Bidding.—One of the leading motives that called this brief article into being was to aid, as far as we might be able to do so, in upholding the dignity and standing of the profession here, and exposing, and deprecating the errors and short comings of some of its members. To err is human, and the best way, probably, to overcome, or abate erroneous practices, is to hold up to the general gaze, such departures from the path of right, and honorable conduct as may be found existing, or occuring frequently among the members of the profession. We are credibly informed that certain practitioners in this city, habitually charge *fifty cents* per visit, and from *five* to *eight* dollars for an obstetrical case; and for other services in the same reduced proportions; and further, that an average of *twenty* to *twenty-five* cents per visit even, is not an infrequent occurrence, with others, where a case of illness is protracted several days! And yet medical societies are numerous, in the city and a "Fee table" of the State Society still exists, (on paper), and is supposed to govern the rates, or charges, of the profession generally, or in this city, at least! That the profession is over crowded *here*, and the supply consequently in excess of the demand for medical attendance, no one will question for a moment; but that men advertising themselves to the community as physicians, are willing to degrade to a point *below* the meanest calling, the grandest profession known to humanity, by asking such pittancies for their services as we have named, surpasses our comprehension completely. It is sincerely to be hoped that those "cheap" practitioners of Baltimore, are alone in their ignoble, if not dishonest work, of undermining and sapping the very foundations of an honorable and noble profession! Such persons as those alluded to, would meet with prompt *expulsion* from *any* of the mechanical trade associations, were they members, and guilty of such nefarious conduct. The opprobious epithet of "Rat," is applied by printers to such unworthy members of their craft, as seek to *under charge* the usual rates for setting type! Shall we use it to designate this class in our profession, as has been already suggested by one or more contemporaries? Or shall the English name of the *Memphitis Americana*, be substituted therefor? Fortunately for our dear old calling, it is believed there are *more men* "righteously" and honorably laboring for its preservation in Baltimore, than were *necessary* to save "the cities of the plains" from merited destruction in the ancient days. We invoke the help of those in an effort to make the "cheap Johns," at least *ashamed* of their *habitual* underbidding? More anon.

